# Thyroid Dysfunction in Patient with Abnormal Uterine Bleeding in a Tertiary Hospital of Eastern Nepal: A Descriptive Cross-sectional Study

**DOI:** 10.31729/jnma.6487

**Published:** 2021-07-31

**Authors:** Bishal Raj Joshi, Shikha Rizal, Shanti Subedi

**Affiliations:** 1 Department of Biochemistry, Nobel Medical College, Biratnagar, Nepal; 2 Department of Obstetrics and Gynaecology, Nobel Medical College, Biratnagar, Nepal

**Keywords:** *hypothyroidism*, *menorrhagia*, *thyroid function tests*

## Abstract

**Introduction:**

Thyroid hormone is known to affect reproductive biology. Abnormal uterine bleeding is one of the common presentations in gynaecology outpatient departments and thyroid dysfunction is known to affect its progression. This study aims to find the prevalence of thyroid dysfunction in diagnosed cases of abnormal uterine bleeding in patients in a tertiary hospital of eastern Nepal.

**Methods:**

A descriptive cross-sectional study was conducted in patients diagnosed with abnormal uterine bleeding in a tertiary care hospital of eastern Nepal from April 2019-March 2020 after taking ethical clearance from the Institutional Review Committee. On basis of inclusion and exclusion criteria, 95 cases of abnormal uterine bleeding were included in the study. A blood sample was taken and a thyroid function test was done by chemiluminescence assay on an automated analyzer. Convenient sampling method was used for sample collection. Statistical analysis was done using Statistical Package for the Social Sciences version 16. Point estimate at 95% Confidence Interval was calculated along with frequency and proportion for binary data.

**Results:**

Out of the total cases of abnormal uterine bleeding, 15 (15.79%) (8.46-23.12 at 95% Confidence Interval) had thyroid dysfunction. Among total cases, 80 (84.21%) were euthyroid. The mean age of the patients was 33±8 years. Among thyroid dysfunction, 9 (60.0%) were hypothyroid, 4 (26.66 %) were subclinical hypothyroid, and 2 (13.33 %) were hyperthyroid.

**Conclusions:**

Thyroid dysfunction was common among patients with abnormal uterine bleeding, with hypothyroidism being the most common type.

## INTRODUCTION

The impact of thyroid hormone on reproductive biology has been studied.^1^ Due to similarity of Thyroid Stimulating Hormone (TSH) to gonadotropins - Follicle Stimulating Hormone (FSH) and, Leutinizing Hormone (LH) and, presence of thyroid hormone receptors on ovaries, thyroid hormones can affect menstrual cycle by direct impact on ovaries or through impact on sex hormone binding globulin (SHBG), prolactin and gonadotropin releasing hormone (GnRH).^1,2^ Abnormal uterine bleeding (AUB) is known to affect 10-15% women of reproductive age globally, with prevalence of 6.2% in Nepal.^3,4^

Hypothyroidism has been found to cause menorrhagia and hyperthyroidism to cause oligomenorrhoea and amenorrhoea.^1^ Most cases of anovulatory bleeding can be treated by hormonal and non-hormonal medicines, thus avoiding surgeries.^5^ Treating thyroid function has shown to improve the menstrual abnormalities.^6,7^

This study aims to find the prevalence of thyroid dysfunction in women of reproductive age with abnormal uterine bleeding in a tertiary hospital of eastern Nepal.

## METHODS

This is a descriptive cross-sectional study that was carried out in department of Biochemistry in collaboration with department of gynaecology at Nobel Medical College, Biratnagar for 12 months from April 2019 to March 2020. Total of 95 diagnosed cases of Abnormal uterine bleeding in gynaecology outpatient department (OPD) were enrolled in the study. Ethical clearance was obtained from Institutional review committee of Nobel Medical College and Teaching Hospital, Biratnagar. Informed consent was taken from patients. Inclusion criteria for selection of patients was diagnosed case of AUB. Exclusion criteria were patients on thyroid medication, under hormonal treatment, contraceptive devices, pregnant patients, bleeding disorders, abortion history within three months, patient with known liver disease and known cases of cancer of genital organs.

Convenient sampling method was used for sample collection. Sample size was calculated by following formula:

n = Z^2^ × (1 - p) / e^2^

  = (1.96)^2^ × 0.2 × (1-0.2) / (0.09)^2^

  = 76

Where,

n = minimum sample size required
Z = 1.96 at 95% Confidence Interval (CI)
p = prevalence of thyroid dysfunction in AUB, 20 %^4^
q = 1-p
e = margin of error, 9%

The sample size was calculated was 76. Adding a 10% non-response rate, the sample size was calculated as 83. We have collected data from 95 participants.

A complete history of age, parity, menstrual history, onset and duration of menstrual problems, volume of blood flow and any other relevant complaints was taken. After case history and clinical examination, urine pregnancy test followed by bleeding time, clotting time, ultrasonography of abdomen and pelvis was done. After all the exclusion criteria ruled out and diagnosis confirmed, patient was prepared for Thyroid function test (TFT).

Approximately 3ml venous blood sample was collected in a yellow capped plain vial, from antecubital vein under strict aseptic conditions following the universal precautions. Centrifugation was done at 3000rpm for 10 minutes and separated serum was stored at -20°C until analysis. Serum triiodothyronine (T3), Thyroxine (T4) and thyroid stimulating hormone (TSH) level were measured by chemiluminescence assay on Advia centaur XP immunoassay analyzer. Normal range for T3, T4 and TSH was respectively 2.5-4.16pg/ml, 0.89-1.76ng/dl and 0.34-5.12IU/ml and thyroid function was interpreted as:

**Table 1:** Values.

Findings	Interpretation
All T3, T4 and TSH within normal range	Euthyroid
T3 <2.5pg/ml, T4 <0.89ng/dl and TSH >5.12IU/ml	Hypothyroid
T3 and T4 within normal range and	Subclinical
TSH >5.12IU/ml	hypothyroid
T3 >4.16pg/ml, T4 >1.76ng/dl and TSH < 0.34 IU/ml	Hyperthyroid
T3 and T4 within normal range and	Subclinical
TSH <0.34IU/ml	hyperthyroid

The data was collected and entered in MS-excel 2013 and analyzed using the Statistical Package for Social Sciences (SPSS) version 16 software. Point estimate at 95% Confidence Interval and descriptive statistics were calculated.

## RESULTS

Out of the total cases of abnormal uterine bleeding, 15 (15.79%) (8.46-23.12 at 95% CI) had thyroid dysfunction. The mean age for patients suffering from abnormal uterine bleeding was found to be 33±8 years and patients from the age group 24-34 years were more common 36 (37.9%) followed by age group 35-44 years 31 (32.6%) (Table 2).

**Table 2:** Age-wise distribution of occurrence of AUB.

Age group (years)	n (%)
15-24	18 (18.9)
25-34	36 (37.9)
35-44	31 (32.6)
≥ 45	10 (10.5)

About 80 (84.21%) were euthyroid (Table 3).

**Table 3:** Thyroid status of study population.

Thyroid status	n (%)
Thyroid dysfunction present	15 (15.79)
Euthyroid	80 (84.21)

**Figure 1:**
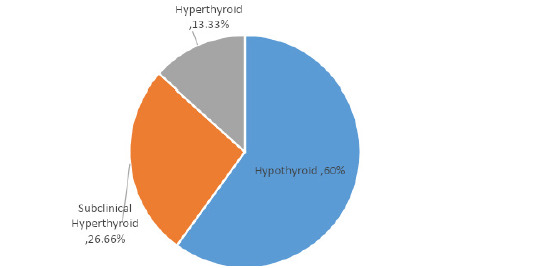
Type of thyroid dysfunction in AUB patients.

Hypothyroidism was most commonamong thyroiddysfunction 9 (60%), followed by subclinical hypothyroidism 4 (26.66%) and hyperthyroidism 2 (13.33%) (Figure 1).

Among the uterine bleeding, menorrhagia was most common 45 (47.37%) followed by metrorrhagia 20 (21.05%), oligomenorrhoea was the least common 3 (3.16%). Thyroid dysfunction commonly occurred in menorrhagia followed by other forms. Among menorrhagia cases, 36 (80%) were euthyroid, 7 (15.5%) were hypothyroid and 2 (4.5%) were subclinical hypothyroid (Table 4).

**Table 3:** Thyroid status in different forms of Abnormal Uterine Bleeding.

	Euthyroid n (%)	Hypo thyroid n (%)	Subclinical Hypo thyroid n (%)	Hyper thyroid n (%)	Total n (%)
Menorrhagia	36 (80)	7 (15.5)	2 (4.5)	0	45 (100)
Metrorrhagia	18 (90)	2 (10)	0	0	20 (100)
Polymenorrhoea	11 (84.6)	0	2 (15.4)	0	13 (100)
Hypomenorrhoea	5 (83.34)	0	0	1 (16.66)	6 (100)
Oligomenorrhoea	2 (66.66)	0	0	1 (33.34)	3 (100)
Menometrorrhagia	8 (100)	0	0	0	8 (100)

**Figure 2:**
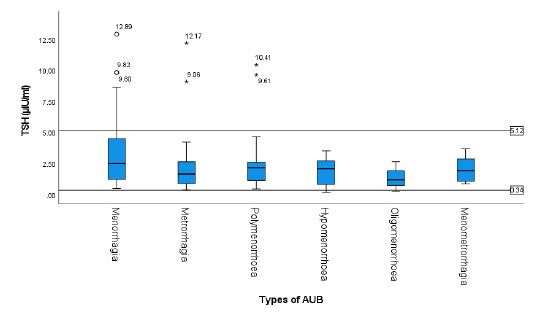
Serum TSH level in different patterns of AUB.

Median TSH (2.12 IQR 1.12-3.60) alone was within the normal range in all types of AUB. But outliers and whiskers of box plot showed considerable cases of menorrhagia, metrorrhagia and polymenorrhoea having TSH above normal range, denoting hypo-functioning of thyroid gland. Box plot also showed cases below normal range in hypomenorrhoea (Figure 2).

## DISCUSSION

Abnormal uterine bleeding is one of the common finding among females of reproductive age. Among the wide spectrum of causes, from structural causes like polyps, leiomyoma etc. to non-structural causes, thyroid dysfunction is found as occult cause which may be readily missed out. Thyroid dysfunction, since being common in women^8^ and has been known to affect all events right from menarche to menopause, cannot be overlooked while treating any forms of menstrual disturbances.^9^

Our study consisted mainly of women from age group 2534 (37.9%) and age group 35-44 (32.6%). In this study, 15.79% of total cases of AUB had thyroid dysfunction. Rest 84.21% of total cases of AUB were euthyroid. Out of cases with thyroid dysfunction, hypothyroid was most common followed by subclinical hypothyroid and hyperthyroid. Hypothyroidism is generally known to cause heavy and irregular menstrual bleeding and hyperthyroidism is generally associated with hypo, oligo and amenorrhoea.^1^ Our finding is similar to studies done in Nepal and India where researchers also found majority of AUB cases associated with euthyroidism followed by hypothyroidism, Subedi, et al.^10^ found 89.3% cases were euthyroid and 9.3% hypothyroid; Thakur, et al.^11^ found 84.9% as euthyroid and 13.9% hypothyroid; Kattel, et al.^4^ found 80% cases euthyroid and 11.1% as subclinical hypothyroid; Bhavani, et al.^12^ found 81% cases as euthyroid and 10% as subclinical hypothyroid; Gowri M, et al.^13^ found 77.6% cases as euthyroid and 17.6% cases as hypothyroid. In contrast according to Ajmani, et al.^14^ 44% of the patients with menstrual disorder had thyroid disorders.

The most common pattern of AUB was found to be menorrhagia was most common 45 (47.37%) followed by metrorrhagia 20 (21.05%), polymenorrhoea 13 (13.68%), menometrorrhagia 8 (8.42%), hypomenorrhoea 6 (6.31%), and oligomenorrhoea 3 (3.15%). This is similar to study done by Kattel, et al.^4^ where menorrhagia (36.7%) was most common complaint among AUB. Bhavani, et al.^12^ found the incidence of menorrhagia to be 54%. Recent similar study in Nepal by Thakur, et al.^11^ also showed menorrhagia to be present among 40.5% cases of AUB. Second most common finding was metrorrhagia in our case which is similar to study done by Muzaffer, et al. (35.4 %).^15^ This is in contrast to study by Thakur, et al.^11^ in which polymenorrhoea was second most common finding.

Observing individual types of AUB, menorrhagia was mostly associated with overt hypothyroidism (15.5%) followed by subclinical hypothyroidism (4.5%). Menorrhagia has been reported to occur in 32-56% of cases of myxedema.^16^ Polymenorrhoea was associated with subclinical hypothyroidism only (15.4%). Oligomenorrhoea and hypomenorrhoea were associated with hyperthyroidism. This finding is similar to study done by Kattel, et al.^4^ Subedi, et al.^11^ and Byna, et al.^17^

TSH can be used as a screening test for every patient visiting gynaecology department with complaint of menstrual disorder. This will provide less financial burden to the patients rather than opting for whole thyroid function test. Median TSH in our study was found to be 2.12IU/L (1.12-3.60) which is in accordance with study done by Khatiwada, et al.^18^ in eastern Nepal who found median TSH to be 2 IU/L (1.0-4.0). Brayshaw, et al.^19^ also reported majority of premenstrual syndrome (35 out of 54 cases) on basis of abnormal TSH test.

This study is a cross sectional study so the cause-and-effect relation of thyroid dysfunction and AUB cannot be elaborated and thus this result cannot be generalized to all patients in our country. Random sampling with more representative sample followed by rigorous study design adjusting the confounding factor would be required in future to further put light on the topic.

## CONCLUSIONS

Our study found substantial patients with AUB suffering from thyroid dysfunction which was similar to previous studies done in similar setting. Hypothyroidism was most common finding in menorrhagia and metrorrhagia and hyperthyroidism was found in oligomenorrhoea and hypomenorrhoea.
